# Robustness of common hemodynamic indicators with respect to numerical resolution in 38 middle cerebral artery aneurysms

**DOI:** 10.1371/journal.pone.0177566

**Published:** 2017-06-13

**Authors:** Øyvind Evju, Jose M. Pozo, Alejandro F. Frangi, Kent-Andre Mardal

**Affiliations:** 1 Center for Biomedical Computing, Simula Research Laboratory, Oslo, Norway; 2 Centre for Computational Imaging & Simulation Technologies in Biomedicine (CISTIB), Department of Electronic and Electrical Engineering, University of Sheffield, Sheffield, United Kingdom; 3 Department of Mathematics, University of Oslo, Oslo, Norway; Technion Israel Institute of Technology, ISRAEL

## Abstract

**Background:**

Using computational fluid dynamics (CFD) to compute the hemodynamics in cerebral aneurysms has received much attention in the last decade. The usability of these methods depends on the quality of the computations, highlighted in recent discussions. The purpose of this study is to investigate the convergence of common hemodynamic indicators with respect to numerical resolution.

**Methods:**

38 middle cerebral artery bifurcation aneurysms were studied at two different resolutions (one comparable to most studies, and one finer). Relevant hemodynamic indicators were collected from two of the most cited studies, and were compared at the two refinements. In addition, correlation to rupture was investigated.

**Results:**

Most of the hemodynamic indicators were very well resolved at the coarser resolutions, correlating with the finest resolution with a correlation coefficient >0.95. The oscillatory shear index (OSI) had the lowest correlation coefficient of 0.83. A logarithmic Bland-Altman plot revealed noticeable variations in the proportion of the aneurysm under low shear, as well as in spatial and temporal gradients not captured by the correlation alone.

**Conclusion:**

Statistically, hemodynamic indicators agree well across the different resolutions studied here. However, there are clear outliers visible in several of the hemodynamic indicators, which suggests that special care should be taken when considering individual assessment.

## Introduction

Unruptured aneurysms constitute a major dilemma for clinicians because of the high prevalence of about 2%, the low annual rupture risk of less than 1%, combined with a high mortality of around 40% associated with rupture [1,2]. For this reason, improved risk assessment is sought to improve treatment and reduce costs. To this end, several indicators representing aneurysm morphology [3–8] and hemodynamics [9–14] have been proposed as biomarkers for aneurysm rupture.

The in vivo measurement of detailed blood flow in intracranial aneurysms is currently infeasible, since the existing methods are invasive and/or too limited in resolution. Because of this, computational fluid dynamics (CFD) have been used to estimate the blood flow in aneurysms and adjacent vessels. However, methods, resolutions, models and hemodynamic indicators vary among studies and confound clinicians [15], and thus, CFD is still not widely considered or used in clinical settings. For instance, the ASME 2012 Challenge [16] displayed significant variations among results obtained from different research groups despite a common surface geometry and flow rate conditions (boundary conditions and mesh varied among the groups). Furthermore, deviation from laminar flow has been observed [17,18]. These studies have spurred significant activity on qualitative assessment of the effect of numerical schemes, software packages and resolution [19–22]. Our motivation with the current study is to address whether resolution plays an important role in quantitative studies. In particular, the velocity and pressure are not the main quantities of interest. Hemodynamic indicators often involve integration and differentiation of velocity and/or pressure in both space and time. From a theoretical point of view, such derived quantities converge at different rates than velocity and pressure, in particular, convergence is often decreased by differentiation and gained by integration.

In the numerous CFD studies on cerebral aneurysms available, a wide range of hemodynamic indicators has been proposed. Most are based on the wall shear stress (WSS), that is, the friction acting on the vessel wall, which may vary considerably both with respect to location, phase in the cardiac cycle, and among patients. Some indicators like maximal or average WSS are motivated by the assumption that the vessels are only able to sustain a certain amount of friction. Others, like oscillatory shear index (OSI), low shear area (LSA), WSS gradients (WSSG), and shear concentration index (SCI) are more directly motivated by the mechanotransduction in the vessel walls. Other indicators attempt to quantify the dissipation or jets in the flow like the viscous dissipation ratio (VDR) and inflow concentration index (ICI). The convergence or sensitivity with respect to resolution, methods and models may vary among these indicators. In [19], the authors highlight the missing or inadequate convergence tests in computational studies. The cause of this is suggested to be the computational time required, but they also point to choice of numerical scheme, with schemes tailored for laminar flow might never capture transitional or turbulent flow patterns. Furthermore, the relationship between the different indicators has been highlighted for being insufficiently discussed [15].

In this study, we have investigated the robustness of common hemodynamic indicators with respect to numerical resolution. We have chosen indicators from two of the most frequently cited studies in the field, namely Cebral et al. [10] and Xiang et al. [14]. From these studies, all hemodynamic indicators significantly correlated with rupture state (p<0.05), were computed. In addition, we computed the wall shear stress gradient (WSSG) and the wall shear stress time derivative (TDWSS) for completeness, in order to capture spatial and temporal variations. We computed all indicators at different refinement levels to investigate the effects of numerical resolution. Finally, we also included the morphological indicators aspect ratio (AR), non-sphericity index (NSI) and volume (V). Correlations among morphological indicators, hemodynamic indicators, and rupture status were investigated to uncover potential surrogates. To limit the amount of variability in the data selection, we limited our study to middle cerebral artery (MCA) bifurcation aneurysms. A total of 38 geometries were studied.

## Methods

3D Rotational Angiography (3DRA) images from 53 patients including a MCA bifurcation aneurysm were selected from the @neurIST [23,24] database. The cerebral vasculature in the region of interest was automatically segmented by a geodesic active region segmentation method [25]. The possible geometrical and topological errors in the resulting vessel surface were manually corrected using the suite @neuFuse [24]. The vasculature of interest was isolated cutting with planes perpendicular to the centerline, and the neck was manually delineated as the surface separating the aneurysm dome from the parent vessels, instead of a single plane [7].

A selection of the suitable geometries was made based on the sufficient length of the vessels present on the segmentation. Geometries where the segmentation did not reach further upstream than the C3 segment of the internal carotid artery (ICA) were excluded from further analysis. This was done to include possible secondary flows initiated around the carotid siphon that propagates downstream, as noted in [26]. In the cases where we had multiple segmentations, the geometry with the longest centerline was selected. A total of 38 geometries were included for further studies, and shown in S1 Fig. A brief summary of the selected dataset is found in Table 1.

**Table 1:** Summary of the selected dataset.

Parameter	Data range
*Ruptured/unruptured*	*13/25*
*Age [years]*	*35–78 (μ = 52*.*8*, *σ = 9*.*2)*
*Female/male [–]*	*28/10*
*Flow rate (ICA) [ml/min]*	*100–382 (μ = 245*, *σ = 62)*
*Flow rate (MCA) [ml/min]*	*58–255 (μ = 134*, *σ = 38)*

The hemodynamic indicators computed include the ones significantly correlated with aneurysm rupture state in either Xiang et al. [14] or Cebral et al. [10]. In addition, the wall shear stress gradient and a wall shear stress time derivative functional are computed for completeness. The exact definitions and significance of each of the indicators computed, are shown in Table 2 and Table 3.

**Table 2:** Definition of computed hemodynamic indicators from literature. For notation, we refer the reader to S1 Table.

Indicator	Abbrev.	Study	Significance	Definition used
*Time- and space-averaged WSS*	*AWSS*	*Xiang et al. [14]*	*R<U (p<0*.*0001)*	$\frac{1}{A_{a}}\int_{\Gamma_{a}}^{}|\bar{\tau }|dS$
*Maximum WSS*	*MWSS*	*Xiang et al. [14] Cebral et al. [10]*	*R<U (p = 0*.*0002) R>U (p<0*.*004)*	$\underset{x\in \Gamma_{a}}{\max}\;\left|\bar{\tau }\right|$
*Oscillatory shear index*	*OSI*	*Xiang et al. [14]*	*R>U (p<0*.*0001)*	$\frac{1}{A_{a}}\int_{\Gamma_{a}}^{}\frac{1}{2}(1-\frac{\left(\bar{\tau }\right)}{\bar{\left(\tau \right)}})\;dS$
*Low shear area*	*LSA*	*Xiang et al. [14]*	*R>U (p<0*.*0001)*	1Aa∫Γa{1,if(τ¯)<0.1AWSS0,otherwisedS
*Viscous dissipation ratio*	*VDR*	*Cebral et al. [10]*	*R<U (p<0*.*0174)*	$\frac{1}{T1-T0}\int_{T0}^{T1}\frac{\frac{1}{V_{a}}\int_{\Omega_{a}}^{}2\frac{\mu }{\rho }{\left|\left|\epsilon \right|\right|}^{2}dV}{\frac{1}{V_{nv}}\int_{\Omega_{nv}}^{}2\frac{\mu }{\rho }{\left|\left|\epsilon \right|\right|}^{2}dV}\;dt$
*Inflow concen-tration index*	*ICI*	*Cebral et al. [10]*	*R>U (p<0*.*004)*	$\frac{1}{T1-T0}\int_{T0}^{T1}\frac{Q_{in}/Q_{pa}}{A_{in}/A_{neck}}\;dt$
*Shear concen-tration index*	*SCI*	*Cebral et al. [10]*	*R>U (p<0*.*049)*	$\frac{1}{T1-T0}\int_{T0}^{T1}\frac{F_{h}/F_{a}}{A_{h}/A_{a}}\;dt$

**Table 3:** Definition of computed hemodynamic indicators added for completion.

Indicator	Abbrev.	Definition used
*Time-derivative WSS*	*TDWSS*	$\frac{1}{A_{a}}\int_{\Gamma_{a}}^{}\bar{\left|\frac{\partial |\tau |}{\partial t}\right|}\;dS$
*WSS gradient*	*WSSG*	$\frac{1}{A_{a}}\int_{\Gamma_{a}}^{}\bar{\left\|\nabla \tau \right\|}dS$

For completeness, we have also included in the study the two morphological indicators most frequently considered [6,7,14] for their high correlation with aneurysm rupture state: aspect ratio (AR) [27] and non-sphericity index (NSI) [4]. Their definition is presented in Table 4, and they were automatically computed from the segmented aneurysm surface and the delineated neck. We have also included the aneurysm volume as size indicator.

**Table 4:** Definition of morphological indicators computed.

Indicator	Abbrev.	Definition used
*Non-sphericity index*	*NSI*	$1-\frac{{\left(18\pi \right)}^{1/3}V_{a}^{2/3}}{A_{a}}$
*Aspect ratio*	*AR*	$\frac{Aneurysm\;depth}{Neck\;width}$

Tetrahedral meshes were generated using VMTK (www.vmtk.org). We generated two sets of meshes to investigate the robustness of the computed indicators with respect to numerical resolution. We varied the resolution depending proximity to the aneurysm domain, increasing the target nodal distance up to a factor 2 furthest from the aneurysm domain. The parent artery had a target nodal distance of 1.25 times the intra-aneurysmal target nodal distance. For small arteries, we reverted to a radius-adaptive sizing method. All meshes included a boundary layer of approximately 0.3 times the target nodal distance. This boundary layer consisted of 4 sublayers, gradually decreasing in thickness by a factor 0.6, resulting in the outermost sublayer to be of an approximate thickness of 0.03 times the target nodal distance.

**Fig 1:**
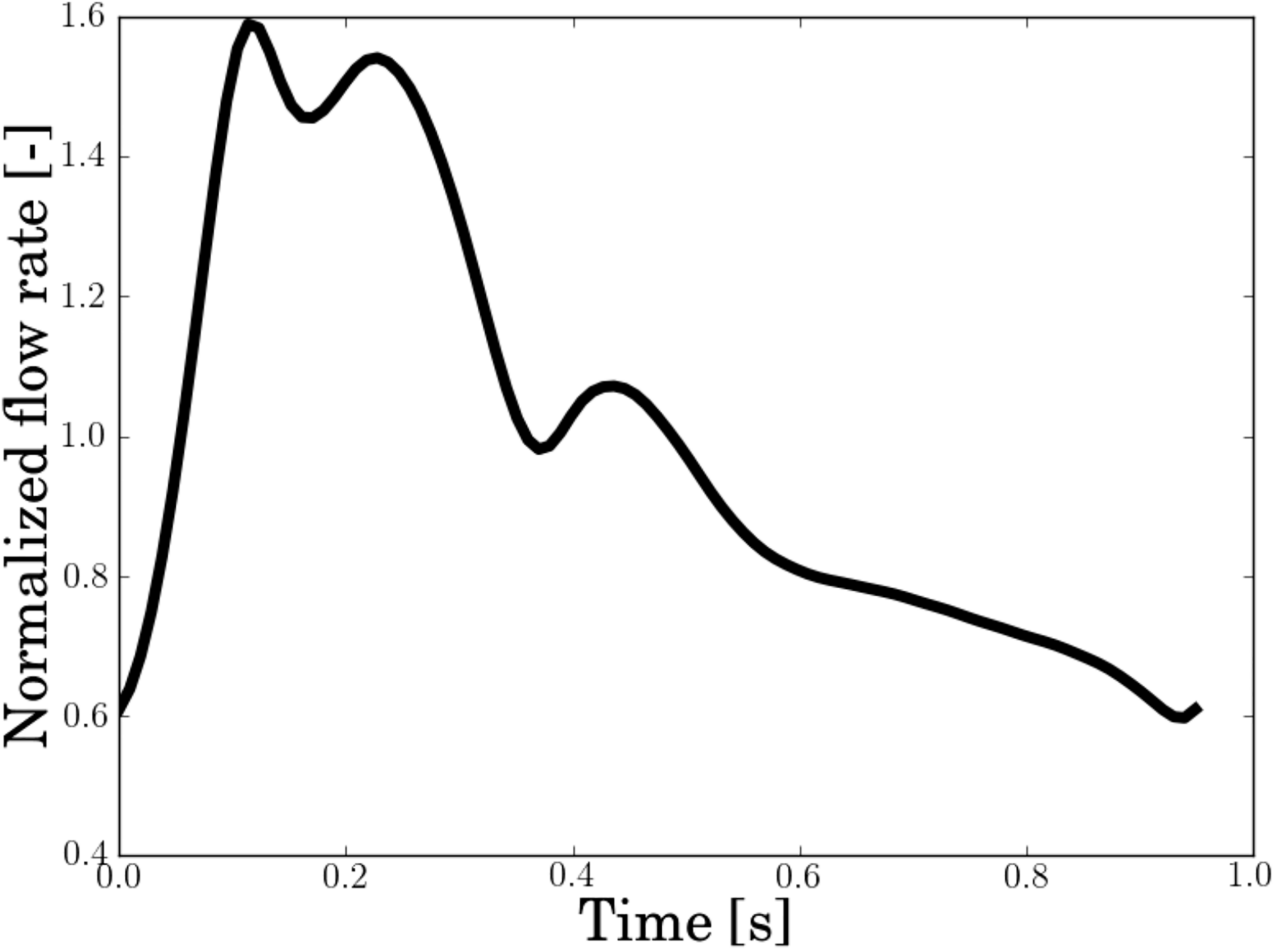
The figure illustrates the differences between the coarsest resolution on the left, and the finest resolution on the right. The clip is done just upstream from the aneurysm of model 1. The black dots represent points at which the velocity is computed.

The coarse meshes were motivated by other studies in the field, limited by the resolution required to resolve the geometry. These consisted of 0.5–1.5 million cells, with an average nodal distance of 0.18mm close to the aneurysm domain, increasing to the double further from the aneurysm. The resolution of the finer meshes used was motivated by the resolutions given in Valen-Sendstad and Steinman [19], and had an average nodal distance of 0.13mm in the near-aneurysm domain. The velocity is approximated using piecewise quadratic polynomials, making the effective mesh resolution 0.065mm. The meshes varied in size from 1.2 to 4.0 million cells. This corresponds to approximately 10–32 million linear elements. Compared to Valen-Sendstad and Steinman [19], our fine spatial resolution is similar to their high resolution (0.065mm to 0.06mm). The coarse meshes are approximately 50% coarser than their normal resolution (0.18mm to 0.12mm), and comparable to the resolution in [10,28]. A comparison of the two refinements is visualized in Fig 1.

By selecting 8000 time steps for the coarse mesh, and 23000 time steps for the fine mesh, we assured the same Courant number for both refinements, which is below 1 for flow velocities up to 1.5 m/s.

All hemodynamic indicators were computed on both sets of refinements, and compared with correlation coefficients, average differences and a Bland-Altman plot [29]. Differences between indicators were calculated globally, that is as the difference in global magnitude, rather than the magnitude of the difference locally. A more comprehensive test was performed on the five first aneurysms of the dataset, where all results were computed on 4 different resolutions. The 2 additional meshes had a resolution between the coarse and the fine meshes. For all resolutions, the velocity was approximated with piecewise quadratic polynomials, and the indicators were computed from the second cycle.

We assume blood to behave as a Newtonian fluid with a dynamic viscosity (μ) of 3.45 mPa s, as justified by Evju et al. [30], and with a density of 1.056 g/mm^3^ (ρ). The walls were assumed to be rigid and impermeable. The inflow boundary conditions were set on the C2/C3 segment as a Womersley profile [31] scaled with the cross-sectional area, as suggested in[32]. The inflow velocity was adjusted to match a physiologically realistic flow rate of 245 ml/min (±61 ml/min) [33]. We matched this with an average inflow velocity of 0.27 m/s, resulting in a standard deviation of 62 ml/min. As we apply the same boundary conditions to all models, the differences in results between the models is only associated with the variations in geometry, as opposed to variations in physiological conditions, such as blood pressure or heart rate.

**Fig 2:**
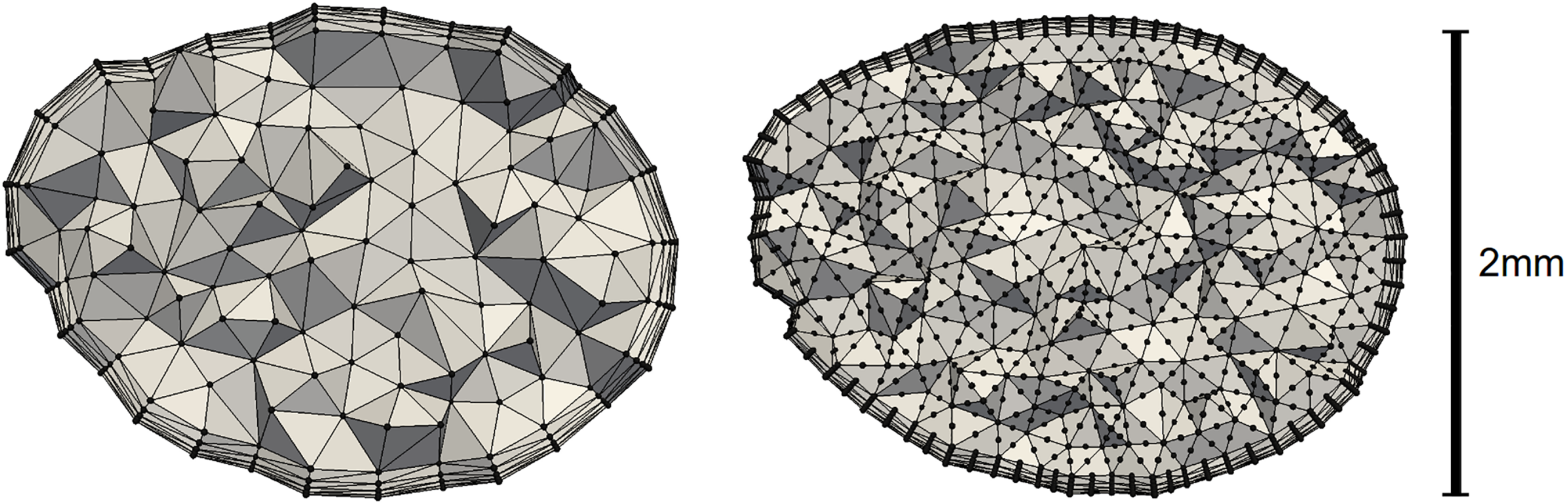
Flow profile used in the simulations.

An average profile is obtained from elderly adults in Hoi et al. [33], measured using cine phase contrast magnetic resonance imaging (PC-MRI) at the C1 segment, with a period of 0.949 seconds (63 bpm). Since we are mainly interested in the flow in the MCA, we reduced the pulsatility by 15% to account for a dampening along the carotid siphon. The dampening has been reported to be in the range -5% to 52% with mean 17.4%. [34] The final dampened flow rate profile is visualized in Fig 2, normalized with 0.27 m/s times cross-sectional area.

On the other arteries included we used pressure conditions to approximately distribute the flow by the principle of minimum work (Murray's law) [35]. More specific, the pressure at artery *i* is set to

$$p_{i}=K\;{\left(\frac{r_{i}^{3}}{\Sigma_{j}r_{j}^{3}}\right)}^{-1}\int_{\Gamma_{i}}^{}u\cdot n\;dS$$

where *r*_*i*_ denotes the radius of artery *i*, Γ_*i*_ is the cross-sectional surface, and ***n*** the outward pointing normal. The summation is over all arteries except ICA, and the constant K is set to 10^9^ $\frac{kg}{m^{4}\;s}$. This outlet condition ensures that the length of the outlets has a minimal effect on the flow division, matching Murray's law at the M2 outlets with an average deviation of -3.2% (±3.2%). These pressure conditions are preferred over velocity outflow conditions, because of the lesser influence on the upstream flow.

The incompressible Navier-Stokes equations were solved using the open source software cbcflow [36] based on FEniCS [37]. The computation of the hemodynamic indicators were done using cbcpost [38]. The solver implementation mimics very closely the one described in [39], and displays second order convergence in both time and space. The scheme is a pressure correction scheme, with a linearization of the convective term, which does not introduce artificial numerical dissipation, as shown in [40]. In fact, the numerical scheme has the same stability property as the continuous equations. At each time step, a tentative velocity is computed using the pressure field from the previous time step. This is then followed by applying the incompressibility constraint to compute a corrected pressure. Finally, the tentative velocity and corrected pressure is used to compute the final velocity.

To determine the correlation with rupture status, we first grouped the results into groups of ruptured and unruptured. We then performed a Shapiro-Wilks test for the normality of the results. Where the null hypothesis of normally distributed data could not be rejected we used a two-tailed t-test. Otherwise, we used a Mann-Whitney U-test. In addition, we performed a Bland-Altman analysis for individualized assessment of the effect of resolution.

## Results

**Fig 3:**
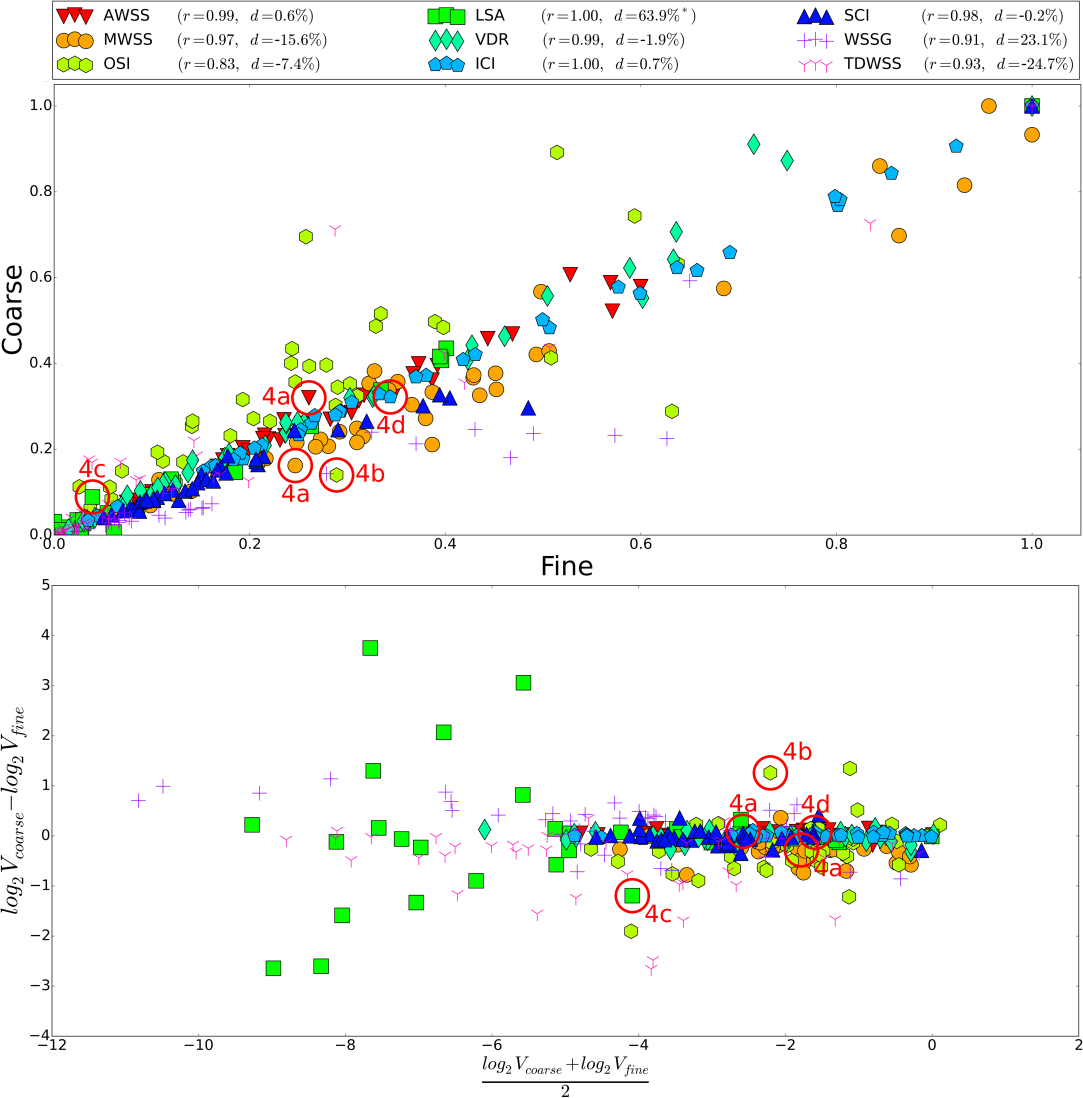
Comparison between the two refinements for all hemodynamic indicators. (A) Correlation plot between coarse and fine refinements. (B) Logarithmic Bland-Altman plot of coarse versus fine (*V*_*coarse*_ *vs V*_*fine*_). r denotes the Pearson correlation coefficient, d the average difference $\left(d=\frac{1}{38}\sum_{i=1}^{38}\frac{V_{coarse}^{i}-V_{fine}^{i}}{V_{fine}^{i}}\right)$. All values are normalized with the max value at the finest refinement. The red circles refer to the cases displayed in Fig 4. *: In 5 cases, LSA was zero for at least one of the two refinements (and less than 0.03 on the other). These are excluded from the Bland-Altman plot and the computation of d, to avoid division-by-zero.

Data from simulations of the 38 aneurysms on two different mesh refinements were analyzed. The correlation between the two sets of simulations performed is shown in Fig 3A. We see that most of the indicators are very well reproduced on the coarsest resolutions, with a few exceptions. That is, AWSS, MWSS, LSA, VDR, ICI, SCI correlate very strongly (r>0.95). The OSI appears to be the most difficult quantity to correctly rank, with a correlation coefficient of 0.834, which is largely caused by a few outliers. Also WSSG (r = 0.910) and TDWSS (r = 0.932) have a number of outliers. We remark that although MWSS demonstrated very strong correlation it is generally underestimated on the coarser refinement with an average deviation of 15.6%.

The variation of the hemodynamic indicators with respect to resolution is shown along the Y-axis of the logarithmic Bland-Altman plot in Fig 3B. We note that the larges variation is in LSA of up to a factor 16. Further, we note that the spread in magnitude of the different indicators for the different aneurysms (X-axis) is large in particular for WSSG, TDWSS and LSA, which is up to three orders of magnitude (2^11^).

Regarding convergence, the global L^2^-norm of the velocity varied by less than 1.2% of time-averaged velocity between the coarse and fine resolution for all aneurysms. The more thorough convergence test on the first five geometries, revealed a difference in the same norm of less than 0.13% between the two finest refinement levels. However, at the two finest refinement levels, the AWSS, VDR, ICI and SCI varied up to 5%. The MWSS, OSI and LSA all showed differences of up to 12%, whereas the WSSG and TDWSS showed differences of up to 40% between the two finest refinements.

**Fig 4:**
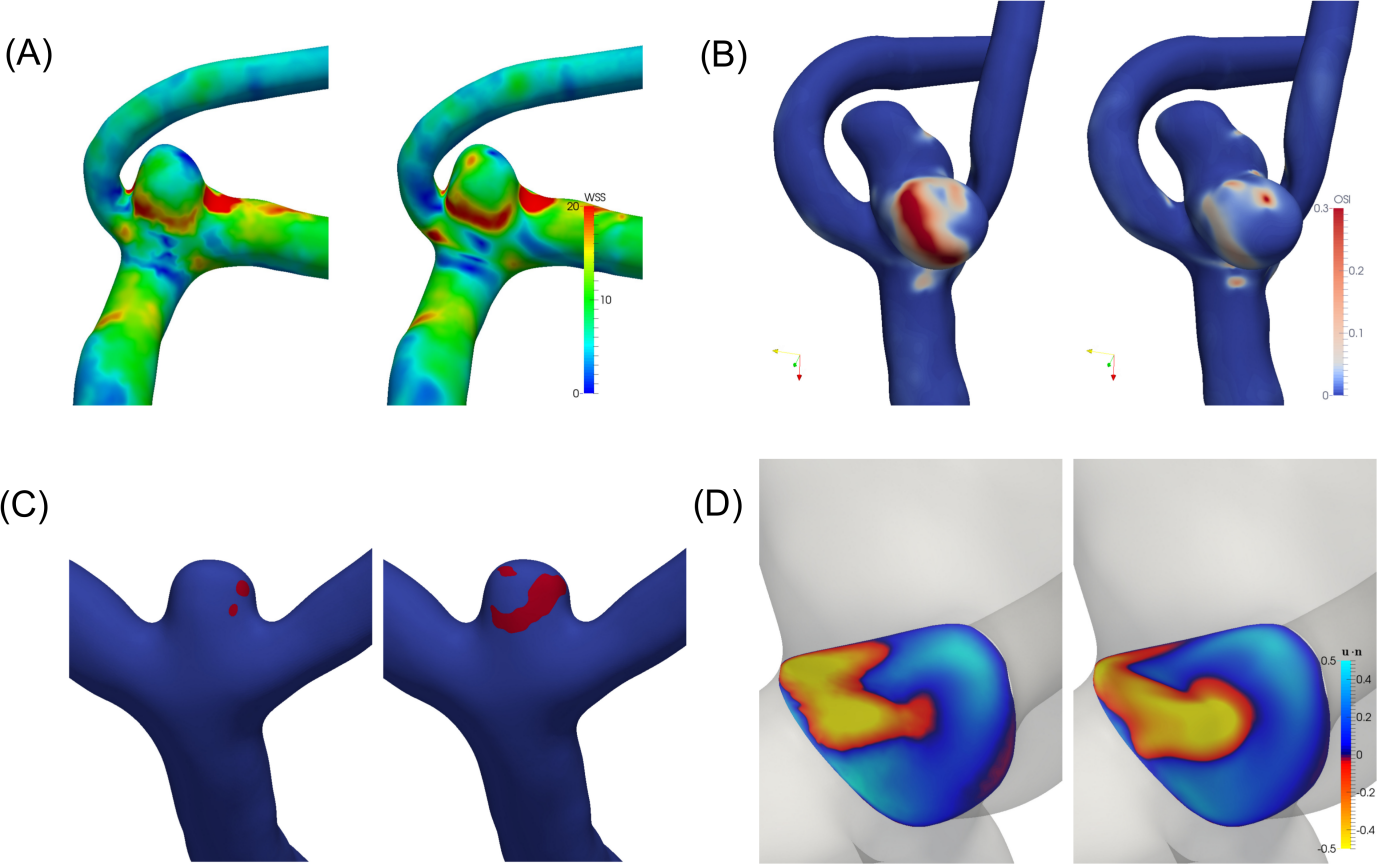
Variability in selected indicators, representative of worst case. Left figures represent the coarse resolution, right figures represent the fine. (A) Shows the WSS fields at different refinements of case 21. (Coarse/fine: AWSS = 9.7/8.9 Pa, MWSS = 43.7/51.8 Pa.) (B) OSI fields at different refinements for case 48 (coarse: 0.028, fine: 0.012). (C) LSA fields (|*τ*| < 0.1 *in red*) at different refinements of case 9 (coarse: 0.033, fine: 0.076). (D) ICI fields at different refinements for case 24 at t = 0.2 (coarse: 1.20, fine: 1.14).

Fig 4 shows a visual representation of worst case scenarios of selected indicators. Fig 4A shows the WSS at coarse and fine resolution in case 21 which is representative for the maximal difference in MWSS and AWSS. Clearly most of the main features are present in the coarse resolutions but there are spots (e.g. at the top) with clear differences. In this case, AWSS was 9.7 Pa and 8.9 Pa and MWSS 43.7 Pa and 51.8 Pa at the coarse and fine resolutions, respectively. Fig 4B shows the differences in OSI fields at coarse and fine resolution in case 48. Clearly, the coarse resolution overestimates the area of local OSI above 0.1 which results in an OSI of 0.028 at the coarse resolution and only 0.012 at the fine resolution. Fig 4C shows the LSA field in case 9, where the coarse resolution clearly underestimates the LSA. This is reflected in the values 0.033 at coarse resolution and 0.076 on the fine resolution. Finally, the ICI field for case 24 is shown in Fig 4D at t = 0.2 where only slight variations between the coarse and fine resolution can be seen. The resulting ICI is 1.20 and 1.14, respectively.

The correlation coefficients between the different indicators shown in Table 5 and Table 6 are less than the correlation between the different resolutions, which is SCI and LSA (r = 0.86). Correlation with P<0.001 was found between AWSS and VDR (r = 0.77), WSSG (r = 0.57), TDWSS (r = 0.53); MWSS and WSSG (r = 070); LSA and SCI (r = 0.86); WSSG and TDWSS (r = 0.63). We note that OSI and ICI have no significant correlation with other indicators at this significance level. Correlation between morphological and hemodynamic indicators was P<0.001 for LSA and AR (r = 0.64); ICI and Volume (r = 0.62); SCI and AR (r = 0.62); AWSS and Volume (r = -0.55). All other correlations had P-values larger than 0.001.

**Table 5:** Correlation coefficients between hemodynamic indicators.

	AWSS	MWSS	OSI	LSA	VDR	ICI	SCI	WSSG	TDWSS
*AWSS*	*1*.*00*^*††*^								
*MWSS*	*0*.*30*	*1*.*00*^*††*^							
*OSI*	*-0*.*10*	*-0*.*17*	*1*.*00*^*††*^						
*LSA*	*-0*.*46*^*†*^	*0*.*19*	*0*.*02*	*1*.*00*^*††*^					
*VDR*	*0*.*77*^*††*^	*-0*.*07*	*0*.*07*	*-0*.*41*^*†*^	*1*.*00*^*††*^				
*ICI*	*-0*.*24*	*-0*.*33*^*†*^	*0*.*41*^*†*^	*-0*.*08*	*-0*.*09*	*1*.*00*^*††*^			
*SCI*	*-0*.*49*^*†*^	*0*.*31*	*-0*.*07*	*0*.*86*^*††*^	*-0*.*51*^*†*^	*-0*.*01*	*1*.*00*		
*WSSG*	*0*.*57*^*††*^	*0*.*70*^*††*^	*0*.*00*	*-0*.*16*	*0*.*23*	*-0*.*33*^*†*^	*-0*.*12*	*1*.*00*^*††*^	
*TDWSS*	*0*.*53*^*††*^	*0*.*31*	*0*.*49*^*†*^	*-0*.*26*	*0*.*35*^*†*^	*0*.*17*	*-0*.*26*	*0*.*63*^*††*^	*1*.*00*^*††*^

^†^: p<0.05

^††^: p<0.001.

**Table 6:** Correlation coefficient between all hemodynamic and morphological indicators.

	AR	NSI	Volume
*AWSS*	*-0*.*46*^*†*^	*-0*.*42*	*-0*.*55*^*††*^
*MWSS*	*0*.*29*	*0*.*22*	*-0*.*19*
*OSI*	*0*.*00*	*0*.*02*	*0*.*07*
*LSA*	*0*.*64*^*††*^	*0*.*43*^*†*^	*0*.*45*^*†*^
*VDR*	*-0*.*51*^*†*^	*-0*.*51*^*†*^	*-0*.*49*^*†*^
*ICI*	*0*.*20*	*0*.*35*^*†*^	*0*.*62*^*††*^
*SCI*	*0*.*62*^*††*^	*0*.*41*^*†*^	*0*.*51*^*†*^
*WSSG*	*-0*.*15*	*-0*.*18*	*-0*.*33*^*†*^
*TDWSS*	*-0*.*21*	*-0*.*11*	*-0*.*22*

^†^: p<0.05

^††^: p<0.001.

Concerning rupture status, summarized in Table 7, we see that all the morphological indicators computed are able to discriminate between the two groups at a statistically significant level. Of these, NSI were the most significant, with higher values in the ruptured group (0.18 to 0.12; p = 0.007). AR was also higher in the ruptured group than in the unruptured group (1.25 to 0.97; p = 0.016). The ruptured aneurysms were also larger, demonstrated with a greater volume (123 to 88mm^3^; p = 0.019). Of the hemodynamic indicators computed, only ICI showed a significant difference between the two groups, with higher values in the ruptured aneurysms (1.64 to 1.29; p = 0.044). MWSS also showed a tendency of higher values in the ruptured group, however not at a significant level (65.2 to 57.2; p = 0.068). The other hemodynamic indicators were not statistically significant, with p-values ranging from 0.131 to 0.896. We remark that large changes between both mesh resolutions were observed in the p-value of OSI (from 0.244 to 0.896) and MWSS (from 0.112 to 0.068). However, none of the indicators changed from not-significant to significant or vice versa with respect to resolution.

**Table 7:** Comparison between the ruptured and unruptured aneurysms for all indicators at both resolutions.

Indicator	Units	Resolution	Ruptured		Unruptured		p-value
			Mean	Std. dev.	Mean	Std. dev.	
*AWSS*	*Pa*	*fine*	*6*.*8*	*6*.*3*	*7*.*3*	*3*.*9*	*0*.*131*
		*coarse*	*7*.*0*	*6*.*5*	*7*.*2*	*3*.*9*	*0*.*190*
*MWSS*	*Pa*	*fine*	*65*.*2*	*32*.*7*	*57*.*2*	*44*.*1*	*0*.*068*
		*coarse*	*51*.*8*	*24*.*8*	*46*.*3*	*30*.*8*	*0*.*112*
*OSI*	*-*	*fine*	*0*.*028*	*0*.*018*	*0*.*029*	*0*.*020*	*0*.*896*
		*coarse*	*0*.*026*	*0*.*014*	*0*.*027*	*0*.*023*	*0*.*244*
*LSA*	*-*	*fine*	*0*.*130*	*0*.*238*	*0*.*069*	*0*.*114*	*0*.*151*
		*coarse*	*0*.*131*	*0*.*234*	*0*.*066*	*0*.*109*	*0*.*144*
*VDR*	*-*	*fine*	*0*.*49*	*0*.*51*	*0*.*63*	*0*.*52*	*0*.*182*
		*coarse*	*0*.*50*	*0*.*53*	*0*.*60*	*0*.*47*	*0*.*190*
*ICI*	*-*	*fine*	*1*.*64*	*0*.*73*	*1*.*29*	*0*.*98*	***0*.*044***
		*coarse*	*1*.*66*	*0*.*74*	*1*.*30*	*0*.*98*	***0*.*041***
*SCI*	*-*	*fine*	*6*.*9*	*8*.*2*	*4*.*5*	*2*.*7*	*0*.*295*
		*coarse*	*6*.*4*	*6*.*7*	*4*.*6*	*2*.*9*	*0*.*306*
*WSSG*	*Pa/mm*	*fine*	*1012*	*1202*	*2138*	*3368*	*0*.*137*
		*coarse*	*1154*	*1516*	*1861*	*2093*	*0*.*124*
*TDWSS*	*Pa/s*	*fine*	*407*	*729*	*400*	*541*	*0*.*235*
		*coarse*	*315*	*577*	*260*	*401*	*0*.*244*
*AR*	*-*	*-*	*1*.*25*	*0*.*43*	*0*.*97*	*0*.*41*	***0*.*016***
*NSI*	*-*	*-*	*0*.*18*	*0*.*05*	*0*.*12*	*0*.*07*	***0*.*007***
*Volume*	*mm*^*3*^	*-*	*123*	*95*	*88*	*143*	***0*.*019***

## Discussion

In this study we investigated the computational robustness of hemodynamic indicators based on two of the most frequently cited quantitative studies in the field [10,14], on a dataset consisting of 25 unruptured and 13 ruptured MCA aneurysms. In addition, we included three common morphological indicators, as well as two hemodynamic indicators for completeness. Coarse and fine resolution simulations correlated very strongly (r>0.95) for AWSS, MWSS, LSA, VDR, ICI, SCI, while OSI (r = 0.83), WSSG (r = 0.91), and TDWSS (r = 0.93) correlated strongly. Although strongly correlated, the important deviations from the identity line observed for LSA (d = 63.9%), TDWSS (d = -24.7%) and WSSG (d = 23.1%) indicates over- or underestimation in the coarser resolution with respect the finer resolution. The inspection of the corresponding logarithmic Bland-Altman plot revealed large variations for the lower values, in particular for LSA. The variation of up to 16 (2^4^) demonstrates that correlation alone is not a sufficient criterion for robustness. From the plot, we see that LSA, WSSG, TDWSS in particular are difficult to accurately compute for lower values.

Our coarse resolution in this study is representative of a normal or high resolution in common CFD analysis of aneurysms. Considering the global L^2^-norm of the velocity, we found only very small changes (<1.2%) between this resolution and our finest resolution. However, even at the finest refinement levels, a detailed convergence analysis of the indicators on five aneurysms showed that not all indicators were converged in the strict sense usually applied to CFD analysis. Only the indicators AWSS, VDR, ICI, and SCI showed a differences of less than 5% between the two finest refinements. MWSS, OSI and LSA showed differences of up to 12%, while WSSG and TDWSS had a difference of up to 40%. Note that as the mesh resolution change, the approximation of the wall geometry also changed slightly. This might contribute to the lack of convergence of several indicators.

Correlation between indicators were weak to none, with the exception of SCI-LSA (r = 0.86), VDR-AWSS (r = 0.77), WSSG-MWSS (r = 0.70), and TWDSS-WSSG (r = 0.63). Correlation between morphological and hemodynamic indicators were weak to none except for LSA-AR (r = 0.64), SCI-AR (r = 0.62), and ICI-Volume (r = 0.62). This suggests that there could be possibilities for surrogates that are more robust, for example the SCI as a surrogate for the less robust LSA.

Only one of the hemodynamic indicators, the ICI, showed a significant difference between ruptured and unruptured aneurysms. The morphological indicators all showed significant differences between the two groups, with NSI as the strongest of the three indicators included. For the other hemodynamic indicators, the tendencies were as expected with basis in the studies they were taken from, but not strong enough to reach statistical significance. The OSI was however an exception from this, where the means were practically identical, and the p-value as high as 0.896. For MWSS, the tendency was towards *higher* MWSS in ruptured aneurysms than unruptured aneurysms, but with a p-value of 0.068, this was not deemed significant. The increased resolution did not change the p-value from significant to not-significant or vice versa for any of the indicators. This suggests that coarse simulations can provide useful information, even though the actual values are not strictly converged.

This study involved 38 MCA aneurysms which is less than the 119 and 210 in Cebral et al. [10] and Xiang et al. [14] and might explain why statistical significance was not obtained. We have also only studied MCA bifurcation aneurysms, which may have different mechanisms related to rupture than aneurysms at other locations. Still, from the coefficient of variation (standard deviation divided by mean) of 0.5–1.83 in Table 7, we can conclude that the hemodynamic variations within aneurysms at this location are substantial. In addition, this study, as comparable studies, is done retrospectively. This further complicates the usage of these indicators as predictors of aneurysm rupture. In particular, it has been shown that the morphology may be significantly affected by aneurysm rupture [41]. Finally, we remark that we have not assumed laminar flow in our simulations and our numerical algorithms were hence not tailored towards such application, using e.g. dissipative or stabilized schemes. The results in particular for OSI, WSSG and TDWSS might have been different if laminar flow was assumed.

The spatial resolution of the quantitative studies considered in [10] and [14] are similar to what we consider coarse resolution. Xiang et al. [14] report 300 000 to 1 000 000 tetrahedral elements, with a hexahedral boundary layer, whereas Cebral et al. [10] report a resolution of 0.1 to 0.2 mm. The temporal resolution is typically much coarser than what we have used, with time steps of 0.001s to 0.01s [19]. This is connected to the solution strategy as mentioned above, an implicit or explicit assumption of laminar flow, and the usage of diffusive schemes or stabilization terms. Other relevant studies such as [12,42–44] either lack information about resolution, or report similar resolutions. In light of the results in this study and recent studies such as [19,22], it seems reasonable to question whether these results are converged in a strict sense. However, using quantitative methods, the results of this study suggest that strict convergence does not alter conclusions based on quantitative analysis.

A detailed grid convergence of 5 aneurysms was done by Hodis et al. [45], where the authors used extrapolation to estimate the grid uncertainty of velocities and average and maximal WSS at peak systole. Our meshes are roughly comparable to their mesh refinements h_4_ and h_1_. They reported grid convergence errors in average WSS of 1–11% and in maximal WSS of 6–15% for the finest mesh. This does not compare directly to our findings, but similar to this study, it highlights the difficulties of strict convergence. They state that each patient-specific model requires individual grid convergence studies. However, as our study shows, this requirement might be unnecessary strict for quantitative studies that considers tens to hundreds of aneurysm models, as the effect of outliers will be diminished.

In Khan et al. [22], the authors study resolution requirements on 3 different aneurysms. They highlight the need for a minimally dissipative solver as more important than grid or temporal resolution. In this study we use the same numerical scheme as the high fidelity solver used by the authors in that study. They state that this high fidelity solver “can tolerate surprisingly coarse resolutions”, and show that in particular AWSS and OSI are properly resolved at spatial resolutions comparable to our coarse resolutions. In our study, we find that for our 38 cases, this still holds true for AWSS, but the OSI seems more difficult to resolve for certain geometries.

## Conclusion

In this study we demonstrate that a quantitative CFD analysis of hemodynamics in cerebral aneurysms, are reasonably robust even though strict convergence in a traditional sense is not obtained. This suggests that the results of the previous quantitative CFD studies such as [10,12,14,40–42], would correlate strongly with properly resolved simulations, although we note that our simulations and scheme may not be representative for all previous studies, which often employ dissipative schemes. However, some hemodynamic indicators such as AWSS, VDR, ICI and SCI are relatively easy to resolve, compared to OSI, LSA, MWSS, WSSG and TDWSS. For individual assessment, special care should be taken that the considered hemodynamic indicators are converged, as there are outliers. Besides ICI, the hemodynamic parameters studied here were unable to discriminate ruptured from unruptured aneurysms.

## Supporting information

S1 Fig Geometries. All geometries used in our computations. Scale is not equal for all images.
(TIFF)

S1 Table A short, but specific definition of the different values used to compute the hemodynamic indicators. The near-vessel domain is defined as the distance from the aneurysm neck of less than 1cm (shortest path, computed using Dijkstra's algorithm).
(DOCX)

## Abbreviations

AR: Aspect ratio
AWSS: average WSS
CFD: Computational fluid dynamics
ICI: Inflow concentration index
MWSS: Maximal WSS
LSA: Low shear area
NSI: Non-sphericity index
OSI: Oscillatory shear index
SCI: Shear concentration index
TDWSS: Time-derivative of WSS
VDR: Viscous dissipation ratio
WSS: Wall shear stress
WSSG: WSS gradient
