# Supplementary figures and images for: Robustness of common hemodynamic indicators with respect to numerical resolution in 38 middle cerebral artery aneurysms
Source: PLoS One. 2017 Jun 13;12(6):e0177566. doi: 10.1371/journal.pone.0177566 (PMC5469453; doi:10.1371/journal.pone.0177566)

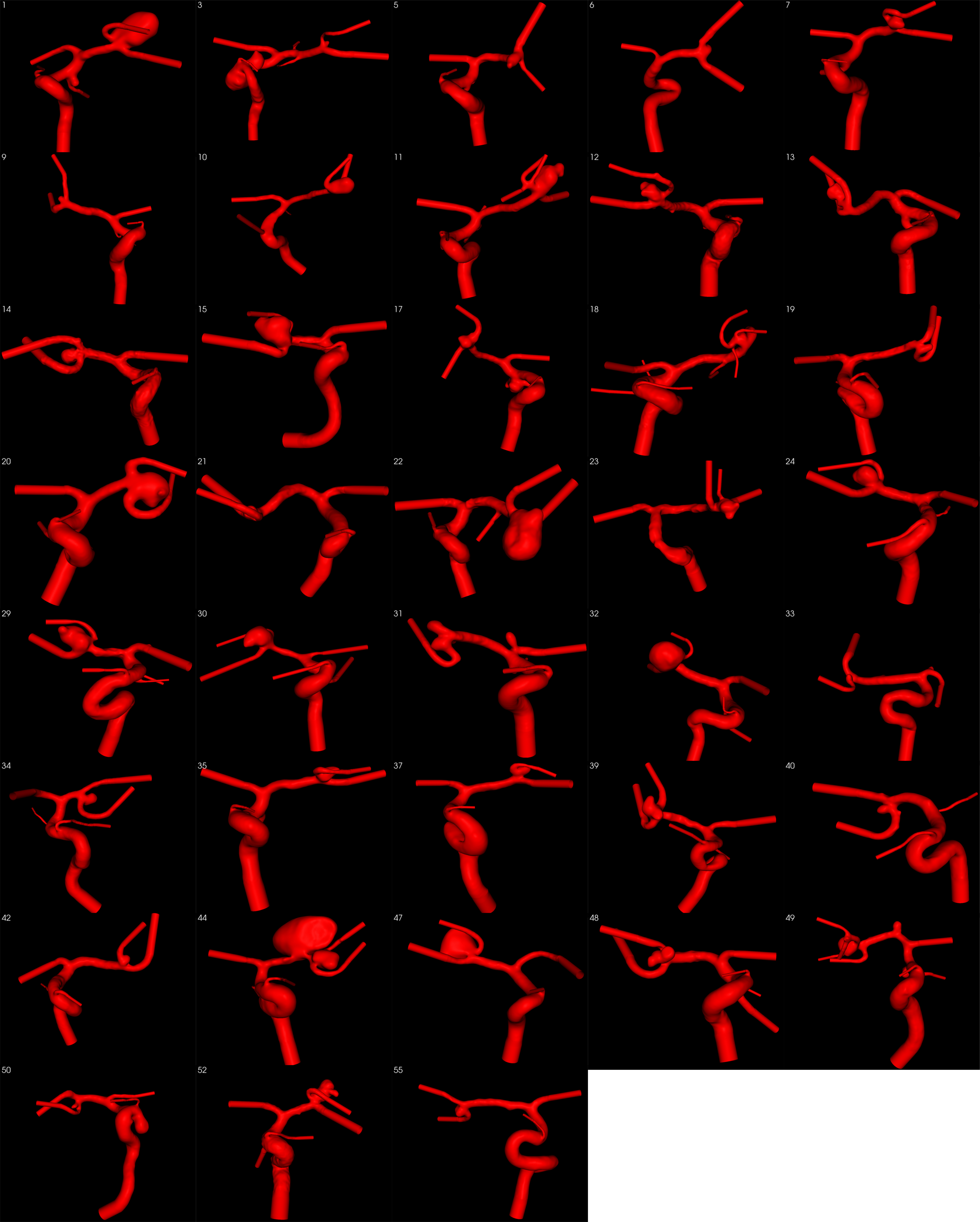

Supplement: S1 Fig — All geometries used in our computations. Scale is not equal for all images. (TIFF) [file pone.0177566.s001.tiff]
