# Supplementary material for: Robustness of common hemodynamic indicators with respect to numerical resolution in 38 middle cerebral artery aneurysms
Source: PLoS One. 2017 Jun 13;12(6):e0177566. doi: 10.1371/journal.pone.0177566 (PMC5469453; doi:10.1371/journal.pone.0177566)
Supplement: S1 Table — The near-vessel domain is defined as the distance from the aneurysm neck of less than 1cm (shortest path, computed using Dijkstra's algorithm). (DOCX) [file pone.0177566.s002.docx]

| T0 | = | Time at start of second cycle | | | | |
| --- | --- | --- | --- | --- | --- | --- |
| T1 | = | Time at end of second cycle |  |  |  |  |
| $\boldsymbol{u}$ | = | Velocity field |  |  |  |  |
| $\boldsymbol{n}$ | = | Outward facing normal on given surface | | | | |
| $\boldsymbol{\tau}$ | = | $\mu\left[ \boldsymbol{n}\cdot\nabla\boldsymbol{u}-\left( \boldsymbol{n}\cdot\nabla\boldsymbol{u}\cdot\boldsymbol{n} \right)\boldsymbol{n} \right]$ | (The wall shear stress vector) | | |  |
| $\epsilon$ | = | $\frac{1}{2}\left( \nabla\boldsymbol{u}+\left( \nabla\boldsymbol{u} \right)^{T} \right)$ | Strain-rate tensor | | |  |
| $\bar{x}$ | = | $\frac{1}{T1-T0}\int_{T0}^{T1} x dt$ | (Cycle-averaged quantity) | | |  |
| $\Omega_{a}$ | = | Aneurysm domain | $\Gamma_{a}$ | = | Aneurysm dome surface |  |
| $V_{a}$ | = | Aneurysm domain volume | $A_{a}$ | = | Aneurysm dome area |  |
| $\Omega_{nv}$ | = | Near vessel domain | $\Gamma_{nv}$ | = | Near vessel domain surface |  |
| $V_{nv}$ | = | Near vessel domain volume | $A_{nv}$ | = | Near vessel domain surface area |  |
| $\Gamma_{neck}$ | = | Neck of the aneurysm | $A_{neck}$ | = | Aneurysm neck area |  |
| $\Gamma_{M2}$ | = | Outflow surface of all M2 arterial segments | | | | |
| $Q_{pa}$ | = | $\int_{\Gamma_{M2}} \boldsymbol{u}\cdot\boldsymbol{n} dS$ | (Flow in parent artery) | | |  |
| $\Gamma_{in}$ | = | $\left\{ \boldsymbol{x}\in\Gamma_{neck} \vert\boldsymbol{u\cdot n>}0 \right\}$ | (Inflow region of neck) | | |  |
| $A_{in}$ | = | $\int_{\Gamma_{in}} dS$ | (Area of flow into aneurysm) | | |  |
| $Q_{in}$ | = | $\int_{\Gamma_{in}} \boldsymbol{u}\cdot\boldsymbol{n} dS$ | (Flow into aneurysm) | | |  |
| $F_{a}$ | = | $\int_{\Gamma_{a}} \boldsymbol{\vert\tau\vert} dS$ |  | | |  |
| $F_{nv}$ | = | $\int_{\Gamma_{nv}} \boldsymbol{\vert\tau\vert} dS$ |  | | |  |
| $\sigma_{nv}^{2}$ | = | $\frac{1}{A_{nv}}\int_{\Gamma_{nv}} \left( \left\vert\boldsymbol{\tau} \right\vert-\frac{1}{A_{nv}}F_{nv} \right)^{2} dS$ | (Std.dev. of shear on near vessel domain) | | |  |
| $\Gamma_{h}$ | = | $\left\{ \boldsymbol{x}\in\Gamma_{a} \vert\left\vert\boldsymbol{\tau} \right\vert> \frac{1}{A_{nv}}F_{nv}+\sigma_{nv} \right\}$ | (Region of high shear) | | |  |
| $A_{h}$ | = | $\int_{\Gamma_{h}} dS$ | (Area of high shear) | | |  |
| $F_{h}$ | = | $\int_{\Gamma_{h}} \vert\boldsymbol{\tau}\vert dS$ | (Total shear in region of high shear) | | |  |
